# Role of Spirometry, Radiology, and Flexible Bronchoscopy in Assessing Chronic Cough in Children

**DOI:** 10.3390/jcm13195720

**Published:** 2024-09-25

**Authors:** Wicharn Boonjindasup, Rahul J. Thomas, William Yuen, Margaret S. McElrea

**Affiliations:** 1 Department of Pediatrics, Faculty of Medicine, Chulalongkorn University, Bangkok 10330, Thailand; wicharn.b@chula.ac.th; 2 Department Respiratory and Sleep Medicine, Queensland Children’s Hospital, South Brisbane 4101, Australia; rahul.thomas@health.qld.gov.au; 3 Australian Centre for Health Services Innovation, Queensland University of Technology, South Brisbane 4101, Australia; 4 Faculty of Medicine, University of Queensland, Herston 4006, Australia

**Keywords:** chronic cough, children, spirometry, radiology, bronchoscopy, pediatric pulmonology, airway assessment, diagnostic tools

## Abstract

Chronic cough in children is a common and multifaceted symptom, often requiring a comprehensive approach for accurate diagnosis and effective management. This review explores the use of spirometry, radiology (chest X-rays and computed tomography (CT) scans), and flexible bronchoscopy in the assessment of chronic cough in children through current guidelines and studies. The strengths, clinical indications, and limitations of each modality are examined. Spirometry, radiology, and in some cases flexible bronchoscopy are integral to the assessment of chronic cough in children; however, a tailored approach, leveraging the strengths of each modality and guided by clinical indications, enhances diagnostic accuracy and therapeutic outcomes of pediatric chronic cough.

## 1. Introduction

Chronic cough is one of the most common presentations in pediatric clinics. In children, chronic cough is typically defined as a cough lasting longer than 4 weeks, rather than the 8-week duration that defines chronic cough in adults. A cough of less than 4 weeks’ duration in children may be due to respiratory infections and may resolve within 4 weeks without further treatment or investigation [1,2,3,4]. The 4-week definition provides the threshold to investigate the possible presence of underlying conditions that are causing the cough, and to provide appropriate treatment where possible [5]. Chronic cough in children is associated with impaired quality of life, missed school days, and multiple physician visits [6]. All international pediatric guidelines support a systematic algorithmic approach to investigating chronic cough in children, as there is high-quality evidence this approach is associated with improved clinical outcomes [2,4,5,7].

## 2. Categorizing Chronic Cough

The assessment of chronic cough includes categorizing cough quality and investigating whether there are any signs suggestive of specific diagnoses [4]. Cough quality can be either ‘wet’ or ‘dry’. Wet cough is a result of excess mucus in the lungs. In children, the term ‘wet cough’ is used rather ‘productive’ as young children do not usually have the ability to expectorate sputum. The excess mucus can be in response to several causes, including infections, structure, foreign bodes, or aspiration. Conversely, dry cough is when no excess sputum is present. This presentation is usually associated with inflammatory, autoimmune, or psychogenic pathologies. In children, the most common diagnosis for presentations with chronic wet cough in the presence of normal lung function and radiology is protracted bacterial bronchitis (PBB) [8]. With dry cough presentations, asthma is the most common diagnosis, especially when exertional dyspnea, expiratory wheeze and/or obstructive spirometry are present [2].

A child presenting with a chronic cough may be further categorized as having “specific cough” or “non-specific cough” [9]. Specific cough is defined as a cough whose characteristics and associated symptoms indicate a specific underlying disease process, while non-specific cough is a diagnosis of exclusion and is a dry cough without known cause [9]. Since specific cough is related to potentially serious underlying conditions, it is important to recognize the signs or pointers of specific cough known as ‘red flags’; for example, cough characteristics such as a wet cough, history of recurrent infections, specific triggers, associated pulmonary and extra-pulmonary signs and symptoms. These ‘red flags’ include severe respiratory symptoms such as hemoptysis and systemic symptoms such as failure to thrive; a full list can be found elsewhere [10]. Hence, children with chronic cough who present with these ‘red flags’ should obtain timely and appropriate management [2,4,5,7].

## 3. Investigating Chronic Cough

There are many investigations to help differentiate between various diseases and conditions associated with chronic cough in children. The cornerstone of investigating chronic cough is performing correct tests at the right time. The clinical decision to order investigations should take these into account.

1. Targeted testing: Accurate diagnosis hinges on selecting appropriate tests based on the child’s history, physical examination, and likely causes. This avoids unnecessary tests and minimizes stress for the child and family.
2. Timeliness: Performing the correct tests at the right stage of the diagnostic process is critical. Early identification of the underlying cause can prevent complications and reduce unnecessary treatment. Conversely, delaying necessary tests may lead to progression of an underlying condition and prolonged discomfort for the child.
3. Cost-effectiveness: Appropriate timing of tests ensures efficient use of healthcare resources. Ordering too many tests can lead to increased costs without improving outcomes, while ordering too few or incorrect tests can lead to missed diagnoses and additional costs from further treatment and testing down the line.

The diagnostic path for children with chronic cough can be challenging and complex, largely due to the broad range of potential causes ranging from common infections to more serious underlying health issues like asthma, allergies, or chronic infections. The challenges also include the overlap of causes, whereby children may have more than one underlying issue contributing to their cough, further complicating diagnosis. Additionally, the ability to describe symptoms may be limited by age, making diagnosis reliant on clinical observation, history from caregivers, and diagnostic testing.

To pinpoint the exact cause without conducting thorough investigations, cough guidelines [2,4,5,7] provide management algorithms to guide the assessment of chronic cough. The assessment includes individual presentation, comprehensive history intake, clinical examination, and appropriate investigations. To improve patient outcomes, investigations must be appropriately ordered, properly conducted, reported in a timely manner, and correctly interpreted. All the guidelines advise chest X-ray (CXR) and spirometry as standard initial evaluation for children with chronic cough [2,4,7]. CXR and, ideally, spirometry are available for primary care clinicians. Beyond this, additional investigations such as chest computed tomography (CT) scans and flexible bronchoscopy with specialist involvement may be indicated.

From a specialist perspective, spirometry, basic and advanced radiology, and flexible bronchoscopy cover a wide spectrum of respiratory system investigations for undifferentiated cough. Henceforth, in this review article, we aim to inform and expand on current guidelines for the role of spirometry, radiology, and flexible bronchoscopy in diagnosing respiratory conditions attributed to chronic cough in children. Initially, we propose the utility of these investigations, as shown in Table 1.

## 4. Role of Spirometry

The non-invasive capacity of spirometry to provide objective data on lung function makes it a valuable tool for initial assessment. Spirometry is a routine part of the assessment in specialist respiratory facilities for all children usually above 6 years of age, and even children from 4 years who are able to perform the correct technique. Healthcare staff performing spirometry must be adequately trained to achieve technically acceptable and repeatable spirometry [30,31]. It is somewhat paradoxical that spirometry is the initial lung function test used to investigate chronic cough given that patients must not cough during a spirometry test. Nevertheless, with patience and skill, the operator should be able to achieve quality spirometry results with most children who present with chronic cough. Spirometry of an acceptable technical standard is helpful for clinical decision-making [32] and the important diagnoses of obstructive and restrictive lung patterns [33].

The key measures in spirometry are forced expiratory volume in one second (FEV_1_), forced vital capacity (FVC), peak expiratory flow (PEF), and the ratio of FEV_1_/FVC. All values need to be compared with appropriate reference values in order to correctly interpret the test results [34]. The shape of the flow–volume curve can also be informative in interpreting spirometry in children, especially in children with chronic cough due to variable or fixed large airway obstruction [33].

An FEV_1_/FVC ratio that is below the lower limit of normal is diagnostic for airway obstruction, and is usually accompanied by a concave expiratory flow–volume curve. Airway obstruction is consistent with several diagnoses presenting with chronic cough in children; these include asthma, bronchiectasis and cystic fibrosis. It should be noted that children with each of these conditions may also have normal spirometry. Spirometry can help confirm a diagnosis of asthma when it demonstrates variable airflow obstruction or significant improvement in airflow obstruction after inhaling short-acting β-agonist bronchodilator. Usually, 200–400 micrograms salbutamol administered by metered dose inhaler and spacer are used as the bronchodilator [11,12,33,35]. GINA and Australian guidelines currently state a significant increase in FEV_1_ to be 12% of baseline value, whereas the most recent ERS/ATS guideline for interpretation has changed the criteria to a 10% increase in %predicted value for either FEV_1_ or FVC [33]. Alternatively, airway hyperresponsiveness can be demonstrated by bronchial challenge testing using either an exercise challenge [36] or inhaled challenge, such as mannitol [37].

Children with PBB usually have normal spirometry; however, spirometry is still helpful in giving reassurance or identifying asthma and tracheomalacia, which may be comorbidities [38]. When tracheomalacia is present, the spirometry expiratory flow–volume curve commonly shows a “knee pattern” and reduced PEF, but the inspiratory phase is normal [39,40].

Children with bronchiectasis (non-cystic fibrosis) are likely to have either normal spirometry or mildly obstructed, depending on disease progression [41,42]. However, spirometry is not a sensitive monitor of short-term changes in clinical signs of bronchiectasis; for example, spirometry values may not change during an exacerbation [42]. Children with undiagnosed cystic fibrosis who present with chronic cough can have varied lung function outcomes on spirometry, either normal, obstruction or restriction, also depending on disease progression [43].

Airway compression such as seen with double aortic arch can cause a fixed extra-thoracic obstruction that gives a characteristic flattened spirometry flow–volume loop and low maximal flows on expiratory and inspiratory phases of spirometry [4,44].

A restrictive pattern on spirometry usually shows a low FVC and preserved or elevated FEV_1_/FVC ratio. The FEV_1_ may be preserved or low depending on disease severity. Confirmation of restriction, i.e., reduced total lung capacity, requires a lung volume measurement, most commonly by body plethysmography [33].

Many spirometry characteristics enable physicians to detect underlying conditions that contribute to chronic cough in children. After initiating treatment for the cause of chronic cough, spirometry is also a practical tool for monitoring response to therapy over time. Improvements in lung function parameters indicate effective management of the underlying condition, while low lung function on spirometry may indicate poor management or predict morbidity and mortality in later life in some respiratory diseases, especially asthma [45,46,47,48], bronchiectasis [42,49] and cystic fibrosis [50].

Whilst there is low-level evidence for the use of spirometry as a non-invasive and inexpensive test, spirometry is recommended as a first line investigation in all children who present with chronic cough.

## 5. Role of Radiology

CXR is the most common radiology method deployed to diagnose conditions affecting the chest and nearby structures. It provides valuable insights into the underlying causes of chronic cough in children, for example, large airway, parenchymal, vascular abnormalities, infections, mediastinal mass, or radio-opaque foreign bodies. CXR findings may also guide further diagnostic tests, such as chest CT scan and flexible bronchoscopy, when a more detailed evaluation is warranted. Because of its provision of useful information, simplicity, relatively low cost and widespread availability, CXR is recommended by all recent international guidelines for use in the initial evaluation for all children with chronic cough [2,4,7,50].

However, CXR may not always provide a definite diagnosis and correlation with clinical history and physical examination. One of the limitations of CXR is its low sensitivity. CXR may not detect small pathologies or early stages of respiratory diseases [51]. Host factors such as younger age, comorbidities and immunologic status may alter the radiologic manifestations. Also, technical errors and a lack of expertise may limit the interpretation of CXR findings in children [52].

To improve the accuracy of assessments of chronic cough in children, additional imaging, especially chest CT scan, is sometimes necessary. CT scanning is fast, non-invasive, precise in acquiring detailed pictures, and helpful in assessing complex or less common causes of chronic cough. The CT scan can provide accurate identification and measurements of structural lung abnormalities. Nevertheless, clinicians should not routinely perform a chest CT scan when there is neither a ‘red flag’ pointer indicating a complex pulmonary condition nor abnormalities on CXR or spirometry [7].

In children with chronic wet cough, a chest CT scan is commonly used to differentiate between PBB and bronchiectasis when there is an inadequate response to treatment [53]. Both PBB and bronchiectasis similarly manifest with chronic wet cough, but bronchiectasis is more likely when there is an ongoing wet cough despite 4 weeks of oral antibiotics [53]. Thus, undertaking a chest CT scan for the definite diagnosis is indicated. In addition, children with multiple PBB episodes are at increased risk of developing bronchiectasis (OR 11.48 (95% CI 2.33–56.50) *p* = 0.003) [54]. Chest CT scan is useful in chronic pulmonary infection for pathology that is not evident on CXR, such as tuberculosis, mycosis, and parasites [55,56,57]. Classic chest CT scan signs and patterns are helpful in guiding further microbiology tests. Moreover, chest CT scans can diagnose rare pulmonary conditions in children, such as interstitial lung diseases, tumors, congenital airway and parenchymal lesions [58].

Another utility of the chest CT scan is in diagnosing tracheomalacia on dynamic airway CT scan, comparing the tracheal lumen at full inspiration with full expiration. Dynamic airway CT scan as an alternative method for diagnosing tracheomalacia is significantly concordant with flexible bronchoscopy [59]. Although flexible bronchoscopy is considered the gold standard, the dynamic airway CT scan is considered acceptable as an objective evaluation when a bronchoscopist is not available.

Despite many benefits, the use of chest CT scans as a routine diagnostic tool is precluded due to cost and accessibility. Patient preparation, and specifically the use of sedation and anesthesia, are sometimes required to achieve a reliable study. Collaboration between clinicians and radiologists is essential for an accurate assessment, including which part of the thorax should be focused on, exact CT scan technique and specific options such as intravenous contrast and inspiratory–expiratory assessment [60]. In addition, concerns about potential cancer risk from a CT scan also need to be weighed against possible benefits [43].

To a lesser extent, ultrasound and magnetic resonance imaging (MRI) are possible modalities in the evaluation of the respiratory system in children. However, they are typically considered adjuncts, given some limitations such as availability at facility, operator dependency, and child’s cooperation. Nevertheless, they can be employed in specific circumstances if the chronic cough could be caused by external compression of the airway from structures, such as a mass or abnormal blood vessels. Ultrasound is recommended as the initial evaluation for certain situations, especially neck ultrasound to evaluate the thyroid gland or detect cervical lymphadenopathy or masses [61]. MRI provides superior soft tissue evaluation of complex and deep neck masses, mediastinal masses, cardiovascular anomalies, and spinal conditions in the non-urgent setting. Despite a lack of clarity regarding its indications, MRI is a radiation-free alternative in patients who require extended follow-up, such as those with cystic fibrosis and mediastinal neoplasms [62].

## 6. Role of Flexible Bronchoscopy

Flexible bronchoscopy, with or without bronchoalveolar lavage (BAL), is a key diagnostic tool that aids in narrowing the diagnostic spectrum of undifferentiated pediatric chronic cough [63,64,65]. BAL is a procedure performed during bronchoscopy that involves washing out the airways and alveolar spaces with normal saline to collect cells, microorganisms, and other substances from the lower respiratory tract. Flexible bronchoscopy and BAL provide information on lung anatomical structures, inflammation, and microbiology, which is valuable for diagnosing and managing the underlying cause of chronic cough.

The role and type of bronchoscopy in children with chronic cough depends on guideline recommendations. Whilst both flexible and rigid bronchoscopies are used in pediatric settings, flexible bronchoscopy is used more commonly in the assessment of chronic cough in children. Both require significant expertise and carry procedural risks despite being described as minimally invasive.

If there is suspicion of a foreign body as the cause of chronic cough, a combined approach should be considered, which would involve rigid bronchoscopy and flexible bronchoscopy. Rigid bronchoscopy has traditionally been considered the standard of care for the removal of foreign bodies in pediatrics [66], based on the ability for better airway control (able to ventilate through the rigid bronchoscope), better optics, and access to larger instruments. However, due to the rigid nature of the instruments, it can only usually reach foreign bodies that are in the proximal tracheobronchial tree (trachea, right main bronchus, left main bronchus). There is also a risk of airway trauma with the rigid instruments. The benefit of the flexible bronchoscope is that the tracheobronchial tree can usually be inspected at the segmental level. Traditionally, flexible instruments have not been as good for the removal of foreign bodies as rigid instruments, but there is emerging evidence regarding the safety of foreign body removal with flexible bronchoscopy (with the advent of cryotherapy) and it ability to fit through the smaller working channel in the flexible bronchoscope [67], as well as its effectiveness when combined with rigid bronchoscopy [68,69].

Flexible bronchoscopy allows for the visualization of the airways using a tip-bending flexible wire and a working channel for interventions. Flexible bronchoscopy is favored over rigid for initial management in most circumstances. This tool is widely used in the evaluation and management of pediatric respiratory conditions due to its versatility and safety. Flexible bronchoscopy can be performed under sedation and local anesthetic, but is most often performed under general anesthesia in children [64,65]. Flexible bronchoscopy also facilitates exploration of the distal airways and dynamic airway assessment (for diagnosis of tracheomalacia and bronchomalacia), and can be performed in children at various stages of development, from neonates to adolescents. Indications for flexible bronchoscopy include a variety of conditions, ranging from exploratory examination, secretion or cytology/microbiology collection, tissue biopsy, and therapeutic interventions such as foreign body removal [63,70,71].

Evidence for flexible bronchoscopy being used effectively in pediatric settings can be seen in the context of medical conditions such as PBB [72]. To achieve a formalized assessment criteria for the severity of bronchoscopically defined bronchitis, a valid scoring tool such as the BScore system can be used to describe key features such as secretion amount, secretion color, mucosal edema, and erythema [73]. In turn, management is changed pending assessment of severity. Another obvious use of flexible bronchoscopy is in examining both static and dynamic structural abnormalities, such as tracheomalacia and trachea–esophageal fistula, known causes of chronic cough [73,74,75].

The guidelines regarding flexible bronchoscopy intervention in children with chronic cough vary. The CHEST guidelines, which are targeted towards developed nations, indicates that flexible bronchoscopy should be utilized under these circumstances: “(1) suspicion of airway abnormality, (2) localized radiology changes, (3) suspicion of an inhaled foreign body, (4) evaluation of aspiration lung disease, and (5) microbiological studies and lavage” [2]. In comparison, the European Respiratory Society does mention flexible bronchoscopy as a tool to inform diagnosis further, but does not specify exact criteria for when bronchoscopy should be performed aside from being important in foreign body aspiration, or when a specific etiology for the cough is suspected [7]. Given the utility and relatively minimal invasiveness and low anesthetic risk of flexible bronchoscopy, more detailed guidelines are needed to derive more comprehensive guidance on when to perform flexible bronchoscopy in children with chronic cough. A randomized controlled trial is currently underway, seeking to clarify the clinical utility of flexible bronchoscopy in children, with the aim of determining the impact of flexible bronchoscopy on quality-of-life scores and management [76].

## 7. Conclusions

In children with chronic cough, performing the correct tests at the right time is critical for arriving at an accurate diagnosis and guiding effective treatment. The diagnostic process requires a careful balance of clinical experience and the judicious use of diagnostic tools, tailored to each child’s individual presentation. While no high-quality evidence has assessed whether spirometry or CXR in children with chronic cough enhanced management, recent guidelines for chronic cough recommend that spirometry and CXR should be undertaken in all children on presentation. Further investigations, especially chest CT scan and flexible bronchoscopy, are valuable, but should be undertaken as appropriate to confirm diagnosis. Additional information on the utility of comprehensive investigations and how to apply these in various settings are warranted in order to mitigate the difficulty of diagnostic path.

## Figures and Tables

**Table 1:** The utility of spirometry, chest X-ray (CXR), chest computed tomography (CT) scan, flexible bronchoscopy, and further investigations to approach possible causes of chronic cough in children.

Suspected Cause of Chronic Cough	Initial Investigations * (Recommended for All)		Additional Investigations * (Recommended as Appropriate)		
	Spirometry	CXR	Chest CT Scan	Flexible Bronchoscopy	Suggested Further Investigation
Asthma [11,12]	To diagnose and monitor airflow obstruction	Likely normal, when no exacerbation	-	-	FeNO, AHR tests, RAST, Therapeutic trial
PBB [13]	Likely normal, but monitor disease progression	To identify comorbidity and/or complication	+	+	Therapeutic trial, Respiratory microbiology
Bronchiectasis [14]	Findings can vary but monitor disease progression	To identify comorbidity and/or complication	+++	+++	Sweat test, immune function tests
Cystic fibrosis [15]	Findings can vary but monitor disease progression	To identify comorbidity and/or complication	+++	+++	Sweat test, genetic tests
Foreign body inhalation [16,17]	May aid in diagnosis: airway obstruction	To identify radio-opaque foreign body and/or air-trapping	+++	+++	Rigid bronchoscopy
Tracheomalacia [18]	May aid in diagnosis: the ‘knee’ pattern on flow–volume loop	Likely normal, but identify comorbidity and/or complication	+	+++	Optional to perform dynamic airway CT scan
Extrinsic airway compression [19]	Aid for diagnosis: airflow obstruction on flow–volume loop	To identify abnormal intra-thoracic structure	+++	++	Barium swallow
Upper airway cough (postnasal drip, rhinitis, rhinosinusitis) [20]	Likely normal	Likely normal	-	-	Allergy tests, therapeutic trial
Tracheo-oesophageal fistula [21]	Not applicable for infant presentation	To identify chronic aspiration	+	+++	Barium swallow, EGDS
GORD [22]	Likely normal, unless co-existent with asthma	To identify chronic aspiration	-	+/-	pH/multimodal monitoring, EGDS
Chronic infections [23,24]	Findings can vary but monitor disease progression	To identify abnormal lung parenchyma and mediastinum	+++	+++	Respiratory microbiology, blood tests
Interstitial lung diseases [25,26,27]	Likely restriction or mixed obstruction-restriction in advanced stages	To identify diffuse lung lesions	+++	++	BAL cytology, tissue biopsy, immunology, genetic tests
Psychogenic cough [28]	Likely normal	Likely normal	-	-	AHR tests, RAST, psychosocial evaluation, and if possible organic cuases are excluded
Cardiovascular-related conditions [29]	Likely normal or non-specific	To identify abnormal intra-thoracic structure	++	+/-	ECG, cardiac catheterization

* The utility of investigation is based on either experts’ opinion or clinical practice guidelines: +++ = definitely beneficial, ++ = probably beneficial, + = possibly beneficial, +/- = unclear benefit, - = unlikely beneficial. FeNO = fractional exhaled nitric oxide, AHR = airway hyperreactivity, PBB = protracted bacterial bronchitis, GERD = gastro-esophageal reflux, EGDS = esophagogastroduodenoscopy, BAL = bronchoalveolar lavage, ECG = electrocardiogram.

## Data Availability

No new data were created in this review article.
